# Acceptability and utilization of a lipid-based nutrient supplement formulated for pregnant women in rural Niger: a multi-methods study

**DOI:** 10.1186/s40795-019-0298-3

**Published:** 2019-07-01

**Authors:** Sheila Isanaka, Stephen R. Kodish, Abdoul Aziz Mamaty, Ousmane Guindo, Mamane Zeilani, Rebecca F. Grais

**Affiliations:** 1 Epicentre, 8 rue Saint Sabin, 75011 Paris, France; 2 Harvard T.H. Chan School of Public Health, 665 Huntington Ave, Boston, MA 02115 USA; 3 Epicentre Niger, Quartier Plateau, Bd Maurice Delens Porte 206, 13330 Niamey, Niger; 4 Nutriset SAS, Route du Bois Ricard, 76770 Malaunay, France

**Keywords:** Acceptability, Utilization, Pregnant women, Lipid-based nutrient supplement (LNS), Hedonic scale, Ready-to-use food

## Abstract

**Background:**

In food insecure settings, it may be difficult for pregnant women to meet increased nutritional needs through traditional diets. A promising new strategy to fill nutrient gaps in pregnancy involves the provision of lipid-based nutrient supplements (LNS). We aimed to assess the acceptability and utilization of a 40 g LNS formulation (Epi-E) with increased micronutrient content relative to the recommended daily allowance among pregnant women in rural Niger.

**Methods:**

We conducted a two-part, multi-methods study among pregnant women presenting to antenatal care in Madarounfa, Niger during two periods (Ramadan and non-Ramadan). Part 1 included two LNS test meals provided at the health center, and Part 2 included a 14-day home trial to simulate more realistic conditions outside of the health center. Open- and closed-ended questions were used to assess organoleptic properties of Epi-E using a 5-point hedonic scale after the test meals, as well as utilization and willingness to pay for Epi-E after the 14-day home trial.

**Results:**

Participants consumed more than 90% of the test meal in both periods. Epi-E was rated highly in terms of overall liking, color, taste and smell during test meals in both periods (median 5/5 for all); only time, mode and frequency of consumption varied between Ramadan and non-Ramadan periods in observance of daily fasting during the holy month.

**Conclusion:**

Epi- E, a 40 g LNS formulation with increased micronutrient content, was highly acceptable among pregnant women in rural Niger, and utilization was guided by household and individual considerations that varied by time period. This formulation can be further tested as a potential strategy to improve the nutritional status of pregnant women in this context.

**Trial registration:**

ClinicalTrials.gov Identifier: NCT02145000. Registered 22 May 2014.

## Background

Nutrient requirements are increased during pregnancy and essential to meet the needs of both the developing fetus and mother. In resource-poor settings with poor food availability or limited dietary quality, it may be difficult for pregnant women to meet optimal nutrient intake through traditional diets alone [1]. Sub-optimal nutrient intake during pregnancy can contribute to adverse pregnancy outcomes, including increased maternal and neonatal mortality, as well as long-term developmental impairment for the child [2–4].

Several strategies are recommended to help meet the higher nutritional requirements during pregnancy, including iron folic acid (IFA) and multiple micronutrient (MMN) supplementation [5]. IFA supplementation alone can reduce anemia and iron-deficiency anemia at term, but is associated with adverse effects from high doses of iron and low compliance [6]. MMN supplementation with IFA has been shown to have positive impacts on neonatal health outcomes, including reduced risk of small-for-gestational age, low birthweight, and stillbirth [7].

Recent evidence suggests that combining MMN with both energy and protein may improve birth outcomes compared to MMN or IFA alone [8, 9]. Therefore, a promising new strategy to meet the nutritional needs in pregnancy involves the provision of lipid-based nutrient supplements (LNS). LNS are micronutrient-rich pastes that are often made of peanuts, oil, sugar and milk powder and require no preparation or refrigeration during home use. First developed in large-quantities (92 g) at therapeutic doses for the treatment of severe acute malnutrition, LNS can also be provided in smaller quantities to meet specific nutrient gaps [10]. The first evidence for LNS use among pregnant women in Ghana and Malawi suggest that a small-quantity dosage (20 g) may be acceptable in these populations [11, 12].

The current study builds on this limited experience by reporting on the acceptability and utilization of LNS among pregnant women in rural Niger. We aimed to assess a 40 g LNS formulation with increased micronutrient content before testing effectiveness in terms of maternal and child health outcomes. Despite the larger dose and increased micronutrient content, we hypothesized that the 40 g LNS would be both acceptable and appropriately utilized among pregnant women in Niger given previous experience with LNS among children in this context and general palatability of the new formulation.

## Methods

### Parent trial

In 2012, a phase III randomized, double-blind, placebo-controlled trial to test the efficacy of the bovine rotavirus-pentavalent vaccine (BRV-PV) began in Madarounfa, Niger (ClinicalTrials.gov Identifier: NCT02145000). Details of the parent trial and study setting have been published elsewhere [13]. In recognition of the need to identify potential boosters of immunogenicity in countries where oral vaccine efficacy is low, a cluster randomized sub-study was designed to identify complementary interventions that may increase vaccine efficacy. One potential mechanism hypothesized to underlie lower immunogenicity observed in some settings is related to the inhibitory effects of poor nutrition on immune development [14]. The role of nutrition in immune development has been reasonably well documented in observational studies, where both macro- and micronutrient deficiencies have been shown to result in the impairment of various components of the immune system [15]. There is also evidence that micronutrient, fatty acid and protein-energy nutrition in early life may play a role in immune development [16, 17], but there have been few randomized studies to evaluate the impact of early nutritional intervention on infant immune development. We therefore proposed to evaluate the effect of prenatal nutrition supplementation on infant immune response as a nutrition-immunogenicity sub-study; the sub-study specifically aimed to test whether daily prenatal nutritional supplementation with LNS or MMN improves infant immune response to three doses of oral vaccine, compared to IFA.

### LNS formulation

A new, 40-g LNS formulation (hereafter referred to as ‘Epi-E’) was developed in collaboration with Nutriset SAS (Malaunay, France) to meet the nutritional needs of pregnant women in rural Niger. It is a modified form of the 20-g LNS developed by the iLiNS Project for supplementation of pregnant and lactating women in Ghana and Malawi [18]. The Epi-E dose was increased from 20 g to 40 g based on findings from Burkina Faso where a higher dose prenatal MMN-fortified food supplement was shown to increase birth length compared to MMN supplementation alone [8]. Epi-E includes iron and folic acid and provides approximately two times the United States Institute of Medicine recommended daily allowance (RDA) of several micronutrients, including twice the recommended amounts of vitamin B complex, zinc, and selenium levels recommended for pregnant women [19] (Table 1). Phosphorus, potassium, magnesium, and calcium levels were provided at the highest levels possible given constraints of taste profile and stability.

**Table 1:** Nutrient composition of Epi-E lipid-based nutrient supplement

Nutrient	United States Institute of Medicine Recommended Dietary Allowance (RDA) for Pregnant Women aged 19–30 years^18^	Epi-E^a^ LNS (40 g daily dose) for Pregnant Women
Energy (kcal)	–	237
Proteins (g)	71	5.2
Lipids (g)	ND	20
LA (Linoleic Acid) (g)	13*	6.9
ALA (α-Linolenic Acid) (g)	1.4*	1.16
Calcium (mg)	1000	559
Phosphorus (free) (mg)	700	400
Potassium (mg)	4700*	400
Magnesium (mg)	350	130
Zinc (mg)	11	30
Copper (mg)	1	4
Iron (mg)	27	30
Manganese (mg)	2	2.6
Iodine (μg)	220	250
Selenium (μg)	60	130
Vitamin A (μg)	770	800
Vitamin B_1_ (mg)	1.4	2.8
Vitamin B_2_ (mg)	1.4	2.8
Niacin (mg)	18	36
Pantothenic acid (mg)	6	7
Vitamin B_6_ (mg)	1.9	3.8
Folic acid (μg)	600	400
Vitamin B_12_ (μg)	2.6	5.2
Vitamin C (mg)	85	100
Vitamin D_3_ (μg)	15	15
Vitamin E (mg)	15	20
Vitamin K_1_ (μg)	90	45

^a^Ingredients list: Non hydrogenated vegetable fat, skimmed milk powder, peanuts, defatted soy flour, sugar, maltodextrin, vitamin and mineral complex, stabilizer: hydrogenated vegetable fat, emulsifier: vegetable lecithin (soy or sunflower), antioxidant

*Adequate intake (AI) reported; everything else presents Recommended Daily Allowance (RDA)

### Recruitment and inclusion criteria

This study was conducted in Madarounfa, a rural department in the Maradi region of Niger. The department comprises several communes, one of which comprises the urban area of Madarounfa. Recruitment took place in June (Ramadan) and again in September (non-Ramadan) 2014 among women presenting for routine antenatal care visits at one study health center. Women at least 18 years of age with a confirmed pregnancy were eligible to participate. Women were excluded if they had a known intolerance to milk or peanuts, or their clinical status at the time of evaluation required inpatient referral. Women who met the inclusion criteria and did not fit the exclusion criteria were given oral and written details of the study and asked to sign or thumbprint the printed informed consent form. After providing consent, participating women were asked to return to the health center the following day. Gestational age of participants was not assessed at the time of enrollment.

### Study design and procedures

This study collected data during two calendar periods: June (Ramadan) and September (non-Ramadan) 2014. Ramadan is the Muslim holy month marked by daily fasting from sunrise to sunset. Pregnant women may be exempt from daily fasting, however household practices may influence individual food preparation and intake during this period. In Niger, where more than 98% of population is estimated to practice Islam, this design allowed for a description of Epi-E acceptability and utilization during two important periods when dietary intake and practices are very different for the majority of the population. In both time periods, a two-part, multi-methods study design was used among two independent samples of participants.

Part I was designed to evaluate Epi-E acceptability through repeated observation of two test meals provided at the health center. Prior to each test meal, trained study staff collected morbidity information from each participant using a self-report of the previous 24 h. Study staff combined Epi-E and locally-purchased ingredients into a fermented maize porridge mixture in a 1:4 ratio. Maize porridge was used in the test meal as it is a common, staple-food preparation available to and often consumed by pregnant women in this context. Each study participant was individually given 50 g of the Epi-E/maize porridge mixture while being observed by a trained interviewer outside of the health center. This amount represented 10 g of Epi-E (approximately two teaspoons) and 40 g of maize porridge (approximately 2–3 tablespoons).

Interviewers weighed the amount of Epi-E/maize porridge mixture that each participant was served and the amount remaining after 15 min. The portion consumed was defined as the difference between the starting and ending volume to the nearest gram. After the test meal, the interviewer asked each participant to rate the characteristics of the Epi-E/maize porridge mixture based on her perceptions of its organoleptic properties, including color, odor, taste, and overall liking, using a hedonic scale with 5 options (1 = *Dislike a lot*, 2 = *Dislike a little*; 3 = *Neither like nor dislike*; 4 = *Like a little*; 5 = *Like* a lot). The 5-point scale was visually depicted using a range of emoticon faces (very unhappy to very happy) that have been previously used to measure food acceptability in illiterate populations [20–22]. Participants were also asked open-ended questions for further explanations and clarifications. The test meal was repeated on two consecutive days to allow women to habituate to the procedures and as a strategy to reduce influence of observation on behaviors.

Part II followed Part I and was designed to simulate more realistic conditions outside of the health center by including a home-feeding trial where participants received a take-home ration of Epi-E to use. A one-day supply of Epi-E included one 40 g sachet to be divided into two meals. Each participant was given a 14-day supply; in the first home trial conducted in June, participants were provided two additional sachets (16 total) in consideration of potential sharing (e.g. distribution of Epi-E among individuals other than the intended beneficiary, including children and other adults within and outside of the household). Participants received oral instructions regarding the appropriate daily dose of Epi-E for consumption, proper storage at room temperature, and the importance of adding it to a meal only after the cooking process.

After completion of the 14-day study period, participants were invited to return to the health center. To estimate compliance, study staff recorded the number of unused sachets returned to the health center with each participant and asked each woman how many sachets were either consumed by the participant or shared with other individuals. A post-trial questionnaire, with both close- and open-ended questions was administered to further assess the incidence of adverse events (e.g. diarrhea, vomiting, fever, reduced appetite, and constipation), as well as the quantity, timing and mode of consumption of Epi-E during the 14-day home trial. In September, interviewers additionally asked participants about their perceptions of Epi-E properties using a hedonic scale, as well as their willingness to use Epi-E again and pay for it in the future after the home trial period. All interviewers provided textual field notes regarding their perception of the product acceptability for further triangulation of data from the interviewers’ perspective.

A sample size of 25 women provided more than 90% power to test the hypothesis that mean consumption in Part 1 was at least 50% of the Epi-E/maize porridge mixture offered, assuming a standard deviation (SD) of consumption of 30% of the amount offered and a true mean of 75%.

### Data analysis

Participant characteristics, including socio-demographic characteristics and self-reported morbidity, as well as results from the Part 1 test meals and Part 2 home trial were summarized using means (SD) for continuous measures, and using counts and proportions for discrete measures. A one-sample t-test was used to assess the difference between the hypothesized and observed mean consumption [23]. Hedonic scale responses rating sensory properties were presented as medians (min, max). Textual responses generated from the open-ended questions underwent an inductive review to identify thematic areas [24]. We considered both their frequency of mention and importance relative to the study aims when deeming emergent themes to be salient. Exemplar quotations were presented in text to illustrate key findings and as corroboration of quantitative results [25, 26]. All quantitative data were double-entered into an EpiData 3.1 database (EpiData, Odense, Denmark) and analyzed using Microsoft 2010 Excel (Microsoft, Redmond, Washington).

This study was conducted according to the guidelines laid down in the Declaration of Helsinki and all procedures involving human subjects/patients were approved by the National Consultative Ethics Committee of Niger, as well as the Western Institutional Review Board. Written informed consent was obtained from all participants.

## Results

Characteristics of participating women are presented in Table 2. Mean (SD) age was 29.5 (7.5) years in June (Ramadan) and 27.9 (7.8) years in September (non-Ramadan), with women reporting a mean of approximately 5 previous pregnancies in both time periods. Very little illness was reported in the 24 h before any test meal, and all participants reported having usual appetites.

**Table 2:** Baseline characteristics and health of participants by day of test meal

Test day, (*n*)	June 2014 (Ramadan)		September 2014 (non-Ramadan)	
	Day 1 (26)	Day 2 (26)	Day 1 (28)	Day 2 (27)
Participant characteristics				
Age, Mean (SD)	29.5 (7.5)		27.9 (7.8)	27.7 (7.8)
Previous pregnancies, Mean (SD)	5.5 (2.9)		5.3 (3.2)	5.2 (3.1)
Reported Illness in previous 24 h, *n* (%)				
Runny nose	0 (0.0)	0 (0.0)	1 (3.6)	1 (3.7)
Cough	4 (15.4)	3 (11.5)	3 (10.7)	1 (3.7)
Difficulty breathing	0 (0.0)	0 (0.0)	0 (0.0)	0 (0.0)
Diarrhea	0 (0.0)	0 (0.0)	0 (0.0)	0 (0.0)
Fever	0 (0.0)	0 (0.0)	0 (0.0)	0 (0.0)
Vomiting/nausea	1 (3.8)	0 (0.0)	0 (0.0)	0 (0.0)
Ear infection	0 (0.0)	0 (0.0)	0 (0.0)	0 (0.0)
Other^a^	0 (0.0)	0 (0.0)	2 (7.1)	0 (0.0)
Usual appetite on test day, *n* (%)	26 (100.0)	26 (100.0)	28 (100.0)	27 (100.0)

^a^*Other* illnesses reported on Day 1 (September 2014) were anemia (*n* = 1) and cold (*n* = 1)

### Test meal results

There was no meaningful variation in results between days (day 1 vs. day 2) or between time periods (June (Ramadan) vs. September (non-Ramadan)). Participants consumed more than 95 and 90% of the 50 g serving provided in under two minutes in June (Ramadan) and September (non-Ramadan), respectively (Table 3). Mean consumption during both test meals was significantly greater than the null hypothesis of 50% consumption (*p* < 0.05). After both test meals, the Epi-E mixture was rated highly, with median responses of ‘5 = *Like a lot*’ in terms of overall liking, color, taste and smell. Participants assigned no score lower than ‘4 = *Like a little*’ to any single category of the hedonic scale. Interviewers also perceived all participants to enjoy Epi-E, noting that women were “*happy and ready to continue using Epi-E*” after the meal.

**Table 3:** Consumption behaviors and attitudes toward Epi-E on day of test meal

	June 2014 (Ramadan)		September 2014 (non-Ramadan)	
	Day 1	Day 2	Day 1	Day 2
No. of participants, *n*	26	26	28	27
Consumption of 50 g Epi-E/maize porridge mixture				
Grams of serving consumed, Mean (SD)	48.56 (1.50)	47.60 (2.18)	45.64 (1.83)	45.78 (1.99)
Average amount consumed of total serving, (%)	97.12	95.20	91.28	91.56
Time (min) taken to consume, Mean (SD)	1.73 (0.96)	1.08 (0.39)	1.54 (0.88)	1.00 (0.68)
Hedonic scale scores of Epi-E, median (min, max)^a^				
Overall liking	5 (5, 5)	5 (5, 5)	5 (4, 5)	5 (5, 5)
Color	5 (5, 5)	5 (5, 5)	5 (4, 5)	5 (5, 5)
Taste	5 (5, 5)	5 (5, 5)	5 (4, 5)	5 (5, 5)
Smell	5 (5, 5)	5 (5, 5)	5 (4, 5)	5 (5, 5)
Interviewer perceptions, *n* (%)				
Interviewer believes participant liked Epi-E	26 (100.0)	26 (100.0)	27 (96.42)	27 (100.0)

^a^Response values ranged from 1 (Dislike a lot) to 5 (Like a lot)

### 14-day home trial results

Participants reported consuming a mean of 14 sachets as prescribed. The mean (SD) number of sachets consumed per day by participants were, however, observed to be higher in June (Ramadan) than in September (non-Ramadan) (1.7 (0.5) sachets/day vs. 1.1 (0.3) sachets/day) (Table 4). These results indicate that not all participants ate exactly 1 sachet per day over the 14-day trial period as prescribed, but all sachets were used and none were reported sold or traded. When probed as to why they consumed the quantity of LNS they did, women in both study periods most frequently reported taking 1 sachet/day in order to “*respect the prescribed dosage*” or because it was “the *instructions that I received*” or “*advice I was given,*” indicating the potential for high compliance with a prescribed dosing with instructions. The only side effects reported were during June (Ramadan) and included 1 case of fever (3.8%) and 1 case of other (“*change in urine color*”) (3.8%).

**Table 4:** Self-reported Epi-E consumption by participants during 2-week home trial period

	June 2014 (Ramadan)	September 2014 (non-Ramadan)
No. of participants, (*n*)	25	23
Sachet utilization at home during 2-week trial period		
No. of sachets received at start of trial period, (*n*)	16	14
No. of total sachets consumed by participant, Mean (SD)	14.0 (0.3)	14.0 (0.2)
No. of sachets consumed by participant per day, Mean (SD)	1.7 (0.5)	1.1 (0.3)
No. of sachets consumed by others, Mean (SD)	2.0 (0.3)	0.0 (0.2)
Any sachets sold or traded, *n* (%)	0 (0.0)	0 (0.0)
Usual amount of Epi-E eaten each time consumed, *n* (%)		
Half sachet	18 (72.0)	2 (8.7)
Full sachet	7 (28.0)	20 (87.0)
More than a full sachet	0 (0.0)	1 (4.3)
Time(s) of day consumed Epi-E^a^, *n* (%)		
Morning	21 (84.0)	18 (60.0)
Midday or Afternoon	2 (8.0)	12 (40.0)
Dinner or Evening	19 (76.0)	0 (0.0)
How usually consumed Epi-E, *n* (%)		
Mixed with food	1 (4.0)	0 (0.0)
Directly from sachet	24 (96.0)	23 (100.0)

^a^The ‘times of day consumed Epi-E’ was calculated as the sum of total reported feeding episodes

In June (Ramadan), 72% of women ate half of one sachet at each serving, with servings most often taken in the morning and again later in the evening. By contrast, in September (non-Ramadan), women more often ate one full sachet each day, in either the morning (60.0%) or midday (40.0%), but never in the evening. Follow-up questions revealed that the preferred time of consumption was dependent on individual-level factors. Participants in both periods indicated that they were “*hungrier*” or had “*big appetites*” in the morning. Others chose when to eat Epi-E based on practical reasons related to work schedules and convenience. One person explained that she specifically chose to eat Epi-E at specific times, “*to keep children from seeing* [her eating it].” During June (Ramadan), participants reported consuming Epi-E early in the morning and/or around dinner or evening time, invoking dietary practices during the holy month for these preferred times, “*Because of the fast, I prefer to take it* [Epi-E] *at dawn and after dinner*.” In both time periods, Epi-E was almost universally consumed directly from the sachet. The most commonly reported reason for doing so was based on flavor. Many women said that they “*like the taste alone, without the mixture*”. Another salient theme was the notion that eating out of the sachet represented “*direct*” consumption, which was explained by participants to be both “*good*” and “*better*” than mixing. Women also cited convenience for using this mode of consumption.

All women in the post-trial assessment reported positive feelings toward the supplement after 14 days of home use, giving Epi-E the maximum score of ‘5 = *Like a lot*’ in terms of overall liking (Table 5). When probed to explain why they liked Epi-E so much, women mentioned they “*have begun to feel energy*” and have gained “*strength*” from using it. Interviewers concurred, noting that all participants in both study periods seemed enthusiastic when discussing their experiences using Epi-E. Field notes from interviewers described the participants to be “*very happy*” and “*satisfied*”, and “*ready to continue*” using it for a longer time period. When asked whether they would be willing to eat Epi-E again, 100% of women replied affirmatively.

**Table 5:** Reported experience and willingness of participants to pay for Epi-E after 2-week home trial period

	September 2014 (non-Ramadan)
No. of participants, (*n*)	23
Hedonic scale scores of Epi-E^a^, median (min, max)	
Overall liking	5 (5, 5)
Color	5 (5, 5)
Taste	5 (5, 5)
Smell	5 (5, 5)
Interviewer’s perception, *n* (%)	
Interviewer believes participant was enthusiastic about Epi-E	23 (100.0)
Willingness to eat Epi-E again, *n* (%)	23 (100.0)
Willingness to purchase Epi-E, *n* (%)	22 (95.70)
Willingness to pay for Epi-E, Mean (SD)	
Amount willing to pay per daily sachet of Epi-E^a^	$0.31 (0.29)
Amount last paid for daily prenatal micronutrient	$0.05 (0.05)
Amount last paid for 1 serving of favorite snack food	$0.31 (0.19)
Willing to pay more for Epi-E than prenatal micronutrient, *n* (%)	23 (100.0)
Willing to pay more for Epi-E than favorite snack, *n* (%)	15 (65.21)

^a^All monetary values reported in USD and based on mid-market exchange rate (1 USD = 516.95 CFA Franc) during month of study (September 2014) www.xe.com

Willingness to pay data were collected from participants in September (non-Ramadan). Nearly all women (95.7%) said they would purchase Epi-E if sold on the market. Women estimated that they would be willing to pay on average (SD) $0.31 (0.29) per sachet, compared to $0.05 (0.05), the average amount last paid for a daily prenatal micronutrient supplement and $0.31 (0.19), the average amount last paid for a serving of their favorite snack food.

## Discussion

Using a two-part, multi-methods design, this study assessed the acceptability and utilization of a new formulation of LNS designed to meet the specific nutrient needs of pregnant women in rural Niger. Findings indicated very high acceptability among the study sample across two time periods. Participants rated Epi-E highly, with positive attitudes toward its organoleptic properties of color, taste, smell as well as an overall liking of the supplement, both immediately following two observed test meals but also after a 14-day home trial. Few side effects were reported, and women expressed an eagerness to continue to use Epi-E and a willingness to pay for it in the future. Acceptability was high in the Ramadan and non- Ramadan study periods, though we observed differing strategies for home utilization during the Ramadan holy month.

LNS has been reported to be highly acceptable for the prevention and treatment of undernutrition in children across various contexts [18, 20–22, 27–34]Ethnographic research using qualitative methods [22, 35–37] and ‘willingness to pay’ studies [38] similarly suggest high acceptability. The high acceptability recorded in this study is most consistent with experience using a 20-g LNS among pregnant and lactating women in Ghana [11]. In this similarly designed study, 64.0% (7/11) of participants gave the 20-g LNS an overall score of 5 (‘Like a lot’) after a 14-day home trial. LNS dose, micronutrient content, as well as social, cultural and economic characteristics of the study settings, however, differed.

Our study provides unique insights into LNS utilization in this population overall, and by time period. We found that participants made decisions about when to eat the supplement on an individual basis, determined by pragmatic household considerations and personal hunger cues that varied importantly by time period. In addition, while all participants were instructed to consume Epi-E as a home fortificant, mixed with local foods, participants preferred consuming Epi-E directly from the sachet. This utilization pattern differs from that found among pregnant women in Ghana, where nearly all participants (91.0%) reported mixing the 20-g LNS with “*porridge*” (73.0%) and “*other foods besides porridge*” (e.g., “*soups, stews, bread, and other traditional dishes*”) [11] and from that found among pregnant and lactating women in Bangladesh, where 83–89% of women mixed the LNS with rice and other foods [39]. It is unclear why such different modes of utilization emerged between settings. However, it may be likely that the mode reported in our study stemmed from participant familiarity with PlumpyNut®, a therapeutic product in the LNS family which is eaten directly from the sachet by malnourished children, and has been used in the Maradi region for over 10 years. It could be attributed to differences in the level of emphasis placed on compliance by study staff in each setting or differences in individual dietary rules or preferences of pregnant women between settings [40, 41]. Consumption direct from the sachet may also be a strategy to limit sharing in a context where food is traditionally shared within the household. When probed, our study participants explained that eating from the sachet just tasted better and was more convenient. Though not prescribed as per study protocol, we observed no adverse implications of this preferred, more convenient mode of utilization.

There are a number of strengths to this study. We first sought to understand acceptability from multiple angles and perspectives, considering the array of indicators to define and assess acceptability. By using both quantitative and qualitative data collection methods on the same topic, our study design included methodological triangulation. By investigating both the attitudes and behaviors of the participants themselves, as well as those reported by the interviewers, we were also able to corroborate findings among different participant types through participant triangulation [26, 42]. The use of repeated observations of test meals on consecutive days reduced the likelihood of reactive behaviors from just a single observation period [43], while repeated observations of the home trial during non-Ramadan and Ramadan time periods provided a more comprehensive understanding of time-dependent dietary behaviors and their potential influence on LNS utilization. These strategies increase our confidence in the positive findings.

This study did have important limitations. First, the home-feeding trial was 14 days long and therefore findings may not reflect long-term acceptability. Ensuring sustained behavior change over time is a particularly difficult aspect of introducing new health practices and may be influenced by household-level utilization factors not assessed in this study [44]. High short-term acceptability observed in this study, however, was accompanied by very few adverse events, suggesting future compliance may be sustainable. High LNS acceptability has recently been shown to be sustained over longer time periods (e.g. up to 12 months) [45], and found to be associated with good adherence during community-based programming [46]. Second, social desirability to provide positive responses may have positively biased participant responses and interviewer field notes [47]. With this in mind, we built in multiple methods to assess LNS acceptability, including the repeated observations, the objective measures of timed and weighed test meals, and the multiple forms of triangulation. Third, the LNS test-meal amount was relatively small (50 g), although if the product were not acceptable based on flavor alone, then it should have been evident even with the small amount provided. Future studies may provide alternative preparations for testing (e.g. alone, mixed with maize meal, mixed with other local foods) to help identify preferences for the mode of utilization relevant for future public health programming. Fourth, the study did not assess participants’ dietary intake or gestational age at enrollment, and did not conduct statistical testing for comparisons other than the primary aim. Finally, these results are context specific, specific to rural Niger where there is high seasonal food insecurity, limited social and economic development [48] and a beneficiary population that has had previous experience with other ready-to-use foods. A relatively smaller sample size was deemed adequate in this specific, homogenous population.

In light of the potential for widespread use of LNS for the prevention and treatment of malnutrition in this setting, future studies are warranted to test and better understand acceptability. Studies among pregnant women with test-meals and home-feeding trials that are conducted over longer time periods, across different seasons, and during special time periods may yield more accurate estimates of acceptability and compliance. Future studies would benefit from data collection at the household-level, where less reactive behaviors may more likely be captured, and from longitudinal follow-up. Such findings are likely to provide a richer perspective of product acceptability and utilization. Larger study samples may also allow researchers to better explore differences in acceptability by participant type.

## Conclusions

This study was the first to assess the acceptability and utilization of a 40 g LNS formulation among pregnant women in Niger. Despite its novel formulation designed to meet the nutrient needs of this vulnerable population with a dose nearly two times the typical given in other interventions, and with a higher-than-usual micronutrient profile, acceptability was high. The mode of utilization varied by household and individual considerations, but did not influence overall consumption. The Epi-E formulation will be tested as a potential strategy to improve the adequacy of nutrient intake and birth outcomes among pregnant women in this context.

## Data Availability

The datasets used during the current study are available from the corresponding author on reasonable request.

## Abbreviations

BRV-PV: Bovine rotavirus-pentavalent vaccine
IFA: Iron folic acid
LNS: Lipid-based nutrient supplement
MMN supplementation: Multiple micronutrient supplementation
RDA: Recommended daily allowance
